# The Skeletal Muscle, the Heart, and the Liver Are the Major Organs of the Accumulation of Nitric Oxide Metabolites after Oral Nitrite Treatment

**DOI:** 10.3390/antiox13030255

**Published:** 2024-02-20

**Authors:** Ana K. Lima-Silva, Macario A. Rebelo, Alessandra C. Barros, Sandra O. Conde-Tella, Jose E. Tanus-Santos

**Affiliations:** 1 Department of Pharmacology, Ribeirao Preto Medical School, University of São Paulo, Ribeirão Preto 14040-900, Brazil; anakarol@usp.br (A.K.L.-S.); conde@fmrp.usp.br (S.O.C.-T.); 2 Department of Pharmacology, State University of Campinas, Campinas 13083-855, Brazil; m219279@dac.unicamp.br (M.A.R.); a272190@dac.unicamp.br (A.C.B.)

**Keywords:** cardiovascular diseases, nitric oxide metabolites, sodium nitrite

## Abstract

Nitrite is a nitric oxide (NO) metabolite, which may be bioactivated to generate NO in vivo and supplement endogenous NO formation, especially in cardiovascular and metabolic diseases. However, it is not known whether treatment with oral nitrite results in the accumulation of NO metabolites in different organs. Moreover, treatment with omeprazole, an inhibitor of gastric acid secretion, severely affects the gastric formation of S-nitrosothiols induced with oral nitrite treatment. However, no previous study has examined whether omeprazole affects the nitrite-induced accumulation of NO metabolites in different organs. This study examined in rats the effects of oral sodium nitrite treatment (15 mg/kg via gavage for 1 or 7 days) associated with omeprazole (10 mg/kg or vehicle) on nitrite and nitrate and nitrosylated species (RXNO) concentrations (measured using ozone-based chemiluminescence methods) assessed in the plasma, aorta, heart, liver, brain, and muscle. While our results showed that NO metabolite accumulation in different organs is not uniform, we found that the skeletal muscle, the heart, and the liver accumulate NO metabolites, particularly RXNO. This response was significantly attenuated by omeprazole in the heart and in the skeletal muscle. Together, these findings may indicate that the skeletal muscle, the heart, and the liver are major reservoir sites for NO metabolites after oral nitrite treatment, with major increases in nitrosylated species.

## 1. Introduction

Nitric oxide (NO) plays a central role in the regulation of various essential physiological processes, including vascular homeostasis [1]. Consequently, a deficiency in this compound is usually associated with numerous pathological conditions, particularly those linked to cardiovascular problems [2,3]. In addition to being produced by NO synthase (NOS) enzymes, NO can also be generated from its chemical by products (NO metabolites), especially nitrite and nitrate, through a complementary pathway known as the nitrate–nitrite–NO pathway [4]. This process involves the intake of nitrate from dietary or other sources and its reduction to nitrite by anaerobic bacteria present in the oral cavity, thus increasing nitrite concentrations in saliva [5]. Following this reaction in the oral cavity, nitrite is swallowed and, in the acidic pH of the stomach, nitrite is further reduced to NO and other NO-related species with important systemic effects [6]. The residual nitrite and nitrate are absorbed by the intestines, and within the circulation, NO and nitrite are oxidized to nitrate, which can either be excreted or re-enter this metabolic pathway [4].

While many studies have shown the biological significance of NO metabolites at physiological levels [7,8], evidence has accumulated supporting the idea that pharmacological approaches aimed at increasing their tissue concentrations can lead to beneficial effects in various pathological conditions [9,10]. In this context, the oral administration of nitrite leads to increases in plasma concentrations of nitrite, nitrate, and S-nitrosothiols, accompanied by corresponding elevations in NO metabolite concentrations across various organs at different time frames [11,12]. Although nitrite has traditionally been regarded as an important reservoir of NO [13,14,15], current evidence suggests that oral nitrite administration stimulates the formation of S-nitrosothiols and other nitrosylated species, which can also release significant amounts of NO in a physiological manner [6,16,17]. This phenomenon potentially leads to the nitrosylation of other enzymes and receptors involved in the regulation of cardiovascular function [18,19], culminating in the accumulation of NO-related species in organs and tissues following a defined administration period. However, no previous study has examined the effects of oral nitrite treatment on the tissue concentrations of NO metabolites.

Omeprazole is a commonly prescribed proton pump inhibitor, which, in addition to rising gastric pH, may cause a variety of other deleterious effects including impaired endogenous NO formation [20,21]. While this effect has been initially attributed to inhibited NOS enzyme activity, it is now clear that omeprazole also interferes with gastric nitrite bioactivation to NO and affects the increases in the concentrations of S-nitrosothiols and other nitrosylated species after oral nitrite administration [22,23]. Consequently, the use of omeprazole, which attenuates nitrite-induced increases in NO-related metabolites generated in the stomach [21], may also affect the increases in tissue storage of NO metabolites after oral nitrite administration. However, no previous study has examined this hypothesis.

In the present study, we hypothesized that the oral administration of nitrite over a span of several days or weeks may result in the accumulation of NO metabolites in tissues. Given that omeprazole affects the mechanisms involved in NO formation [6,20,21], we have also examined whether this drug modifies oral-nitrite-induced modifications in the concentrations of NO metabolites in various tissues.

## 2. Materials and Methods

### 2.1. Animals and Drug Treatment

The animals used in the present study were handled according to the guiding principles published in the National Institutes of Health Guide for Care and Use of Laboratory Animals, and this study followed the guidelines of the Ribeirao Preto Medical School at the University of Sao Paulo. Male Sprague Dawley rats (250–300 g) were obtained from the colony at University of Sao Paulo and maintained on a 12 h light/dark cycle at room temperature (22–25 °C) with free access to standard rat chow and water.

The experiments were designed to assess the concentration of NO metabolites in the tissues and organs after chronic treatment with sodium nitrite. Sprague Dawley rats were divided into four distinct groups (n = 7 or 9 per group). Control rats received water or sodium nitrite (daily 15 mg/kg via gavage) [12] for 1 or 7 days. Omeprazole-treated rats (daily 10 mg/kg; intraperitonially) [22] received the same doses of sodium nitrite or water. The animals were euthanized at the end of each treatment period (1 or 7 days). The reagents were purchased from Sigma Chemical Co., (St. Louis, MO, USA) and all solutions were prepared immediately before use.

### 2.2. Measurement of Gastric pH

To assess the effect of omeprazole, gastric pH measurement was performed. The abdominal cavity was opened, and the pyloric portion of the stomach was properly secured. Subsequently, an incision was made in the lower portion of the esophagus, and a pH electrode (Jenway, Model 3510, Cole Palmer, Bunker, CT, USA) was insert.

### 2.3. Tissue Harvesting and Homogenization

Prior to tissue harvesting, the rats were anesthetized with ketamine (100 mg/kg) and xylazine (10 mg/kg) [24]. It is important to note that blood and tissue samples were collected precisely 6 h after the last oral nitrite administration. Blood was collected into heparin-containing tubes via puncture of the left ventricle and centrifuged for 4 min at 1000× *g* at 4 °C. Plasma was separated, and aliquoted into amber tubes containing 10 mM N-ethylmaleimide (NEM) and 2 mM diethylenetriaminepentaacetic acid (DTPA) [7] for the preservation of nitrosylated species. After blood collection, a catheter was inserted into the aorta and the rats were perfused with a PBS pH 7.4 solution containing NEM (10 mM) and DTPA (2 mM) to prevent the destruction of nitrosylated species [7]. At the end of perfusion, we harvested the heart, aorta, brain, and liver, which were immediately frozen in liquid nitrogen and stored at −70 °C for a further biochemical analysis. Tissue samples were macerated in a glass macerator with PBS, pH 7.4, with NEM/DTPA [25].

### 2.4. Measurement of Nitrite and Nitrosylated Species (RXNO) Concentrations

Tissue homogenates or plasma aliquots were analyzed in duplicate for their nitrite and RXNO levels using an ozone-based reductive chemiluminescence assay as previously described [25]. For this purpose, 50 µL of samples was injected into an acidified tri-iodide solution, and purged with nitrogen in line with the NO analyzer (Sievers Model 280 NO analyzer, Boulder, CO, USA). To measure RXNO, 200 µL of plasma samples or 300 µL of the tissue homogenate was treated with acid sulfanilamide (10% sulfanilamide in 1 M/L HCl) for 5 min prior to injection into the acidified tri-iodide solution, and purged with nitrogen in line with the NO analyzer. The concentrations of nitrite and RXNO in the tissues were normalized by weight of macerated tissue.

### 2.5. Measurement of Nitrate Concentrations

Tissue homogenates or plasma aliquots were analyzed in duplicate for their nitrate levels using an ozone-based reductive chemiluminescence assay as previously described [12]. Nitrates were measured via an injection of 20 µL of the plasma or 50 µL of the tissue homogenate into a solution of vanadium (III) in 1 M hydrochloric acid at 95 °C. The concentrations of nitrate in the tissues were normalized by weight of macerated tissue, whereas plasma concentrations were normalized by the volume of plasma.

### 2.6. Statistics

The results (mean of duplicates) are expressed as means ± S.D. Comparisons between the groups were analyzed with a two-way analysis of variance (Version 6 of GraphPad Prism Software). A probability value of *p* < 0.05 was considered significant.

## 3. Results

### 3.1. Time-Dependent Changes in Plasma NO Metabolite Concentrations

**Figure 1:**
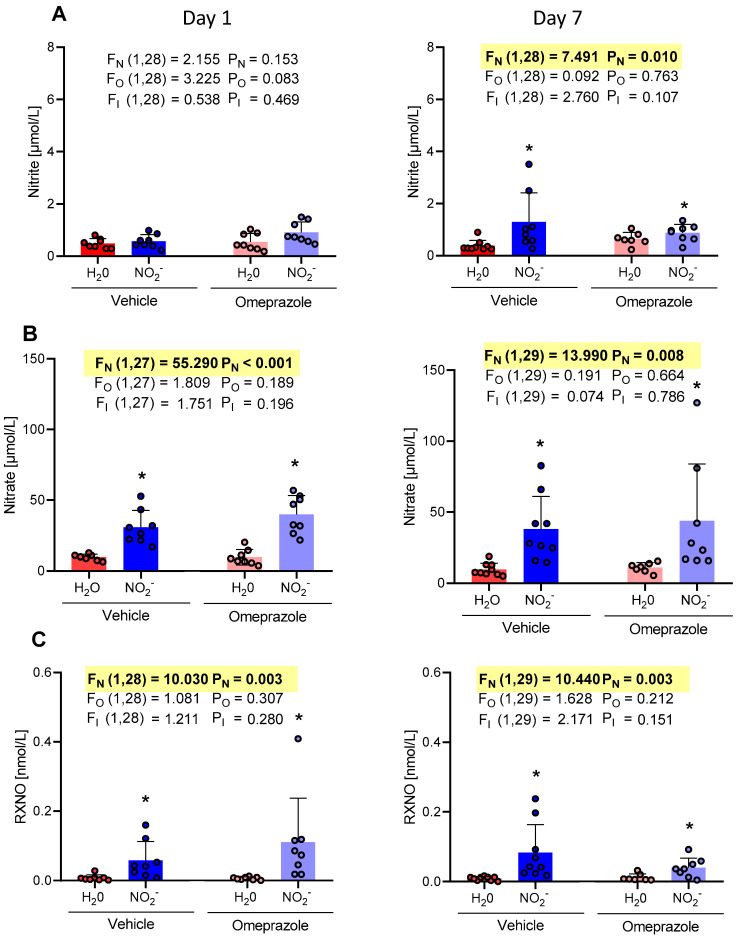
Changes in the concentrations of nitrite, nitrate, and RXNO measured in plasma samples on day 1 and on day 7 of treatment with sodium nitrite (15 mg/kg) or water via gavage in both vehicle- or omeprazole (10 mg/kg)-treated rats. Blood samples were collected precisely 6 h after the last dose of oral nitrite. (**A**–**C**) display the concentrations of nitrite, nitrate, and RXNO, respectively. Data presented as mean ± S.D. (n = 7–9/group). The values of F correspond to the F-statistics for each factor (F_N_ for nitrite factor, F_O_ for omeprazole factor, and F_I_ for the interaction between factors). * *p* < 0.05 versus respective H_2_O group.

Plasma nitrite concentrations (Figure 1A) increased only on the 7th day of treatment (NO_2_^−^ vehicle group exhibited an average of 1.29 µMol/L compared to 0.21 µMol/L in the H_2_O vehicle group), whereas the concentrations of nitrate and RXNO (Figure 1B and Figure 1C, respectively) increased from the first day and after 7 days of treatment with oral nitrite. On the first day of treatment, the NO_2_^−^ vehicle group exhibited an average nitrate concentration of 30.72 µMol/L compared to 9.60 µMol/L in the H_2_O vehicle group. Similarly, the concentrations in the omeprazole NO_2_^−^ group demonstrated an average of 39.97 µMol/L vs. 5.65 µMol/L in the omeprazole H_2_O group. By the 7th day of treatment, the NO_2_^−^ vehicle group showed an average concentration of 38.03 µMol/L vs. 9.75 µMol/L in the H_2_O vehicle group. The omeprazole NO_2_^−^ group exhibited an average concentration of 43.83 µMol/L vs. 11.10 µMol/L in the omeprazole H_2_O group (Figure 1B). Similarly, on the first day of treatment, the NO_2_^−^ vehicle group exhibited an average RXNO concentration of 0.05 µMol/L compared to 0.003 µMol/L in the H_2_O vehicle group. RXNO concentrations in the omeprazole NO_2_^−^ group demonstrated an average of 0.11 µMol/L vs. 0.009 µMol/L in the omeprazole H_2_O group. By the 7th day of treatment, the NO_2_^−^ vehicle group showed an average concentration of 0.08 µMol/L vs. 0.003 µMol/L in the H_2_O vehicle group. The omeprazole NO_2_^−^ group exhibited an average concentration of 0.02 µMol/L vs. 0.005 µMol/L in the omeprazole H_2_O group (Figure 1C). Treatment with omeprazole did not affect the concentrations of NO metabolites measured in plasma samples.

### 3.2. Time-Dependent Changes in Tissue Aortic NO Metabolite Concentrations

The metabolites of nitric oxide can be detected in different tissues and organs, but little is known about the accumulation of these metabolites over time. To investigate variations in tissue concentrations of NO metabolites, we measured the concentrations of NO metabolites in various tissues.

**Figure 2:**
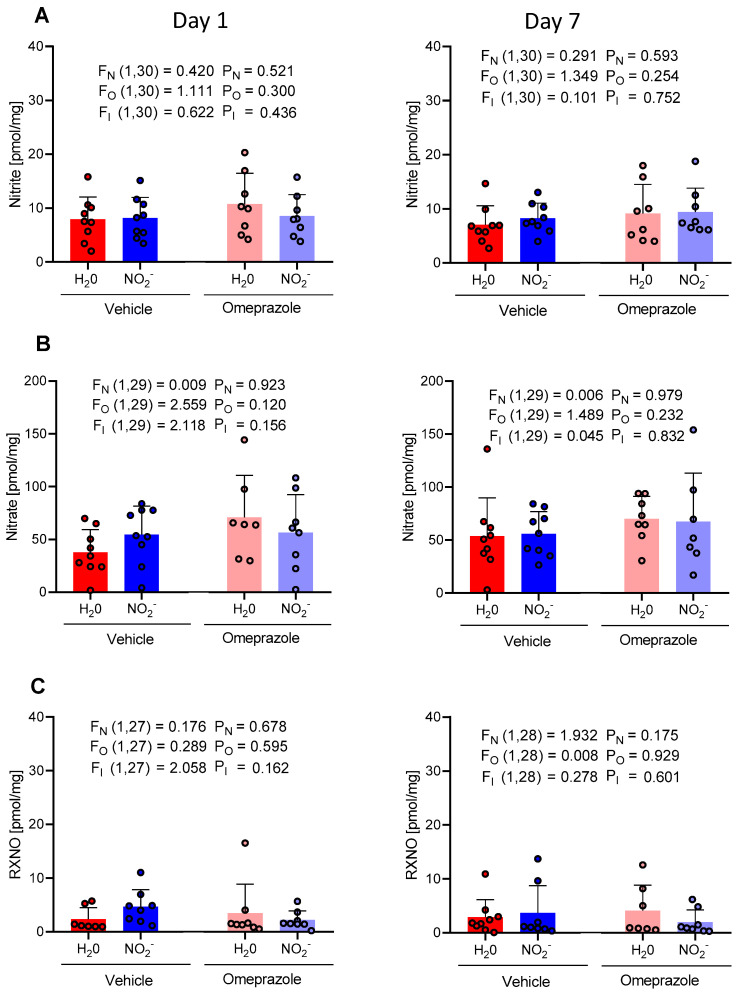
Changes in the concentrations of nitrite, nitrate, and RXNO measured in the aortas on day 1 and on day 7 of treatment with sodium nitrite (15 mg/kg) or water via gavage in both vehicle- or omeprazole (10 mg/kg)-treated rats. Blood samples were collected precisely 6 h after the last dose of oral nitrite. (**A**–**C**) display the concentrations of nitrite, nitrate, and RXNO, respectively. Data presented as mean ± S.D. (n = 7–9/group). The values of F correspond to the F-statistics for each factor (F_N_ for nitrite factor, F_O_ for omeprazole factor, and F_I_ for the interaction between factors).

The concentrations of nitrite, nitrate, and RXNO measured in aortic tissue (Figure 2A, Figure 2B, and Figure 2C, respectively) did not change after 1 or 7 days of treatment with oral nitrite. Moreover, treatment with omeprazole did not affect those concentrations.

### 3.3. Time-Dependent Changes in Brain NO Metabolite Concentrations

**Figure 3:**
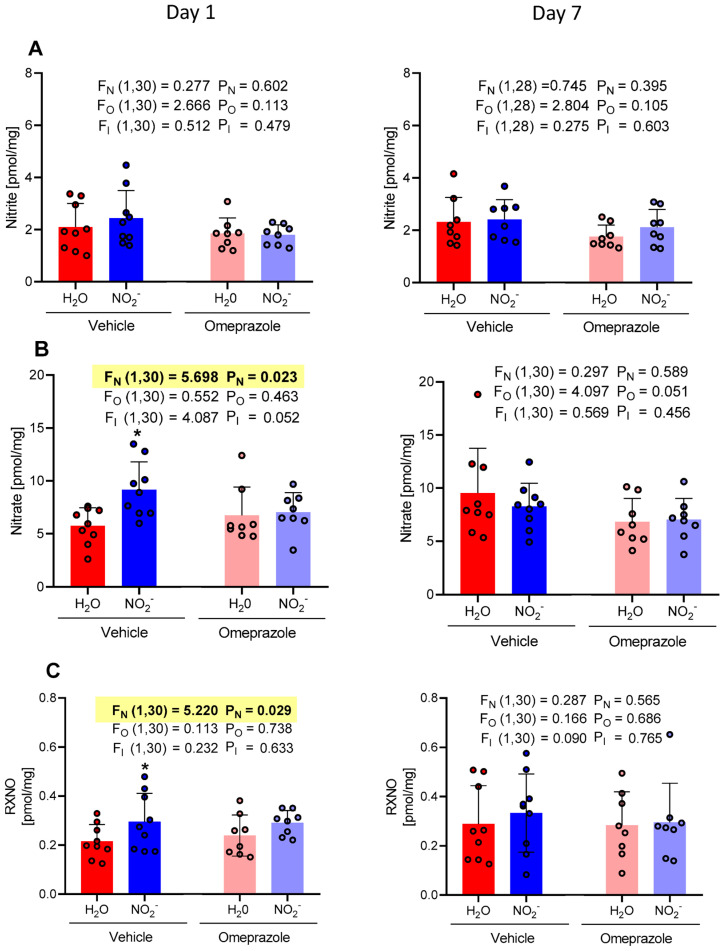
Changes in the concentrations of nitrite, nitrate, and RXNO measured in the brain on day 1 and on day 7 of treatment with sodium nitrite (15 mg/kg) or water via gavage in both vehicle- or omeprazole (10 mg/kg)-treated rats. Blood samples were collected precisely 6 h after the last dose of oral nitrite. (**A**–**C**) display the concentrations of nitrite, nitrate, and RXNO, respectively. Data presented as mean ± S.D. (n = 7–9/group). The values of F correspond to the F-statistics for each factor (F_N_ for nitrite factor, F_O_ for omeprazole factor, and F_I_ for the interaction between factors). * *p* < 0.05 versus respective H_2_O group.

While treatment with oral nitrite did not affect brain concentrations of nitrite after 1 or 7 days of treatment with oral nitrite (Figure 3A), we found increased nitrate and RXNO concentrations after 1 day (but not 7 days) of treatment (Figure 3B and Figure 3C, respectively). On the first day of treatment, the NO_2_^−^ vehicle group exhibited an average nitrate concentration of 9.16 pmol/mg compared to 5.74 pmol/mg in the H_2_O vehicle group. The concentrations in the omeprazole NO_2_^−^ group demonstrated an average of 7.02 pmol/mg vs. 6.73 pmol/mg in the omeprazole H_2_O group (Figure 3B). Additionally, the RXNO concentrations on the first day of treatment in the NO_2_^−^ vehicle group had an average of 0.29 pmol/mg compared to 0.21 pmol/mg in the H_2_O vehicle group. The concentrations in the omeprazole NO_2_^−^ group demonstrated an average of 0.29 pmol/mg vs. 0.23 pmol/mg in the omeprazole H_2_O group (Figure 3C). Again, treatment with omeprazole did not affect nitrite-induced effects.

### 3.4. Time-Dependent Changes in Heart NO Metabolite Concentrations

**Figure 4:**
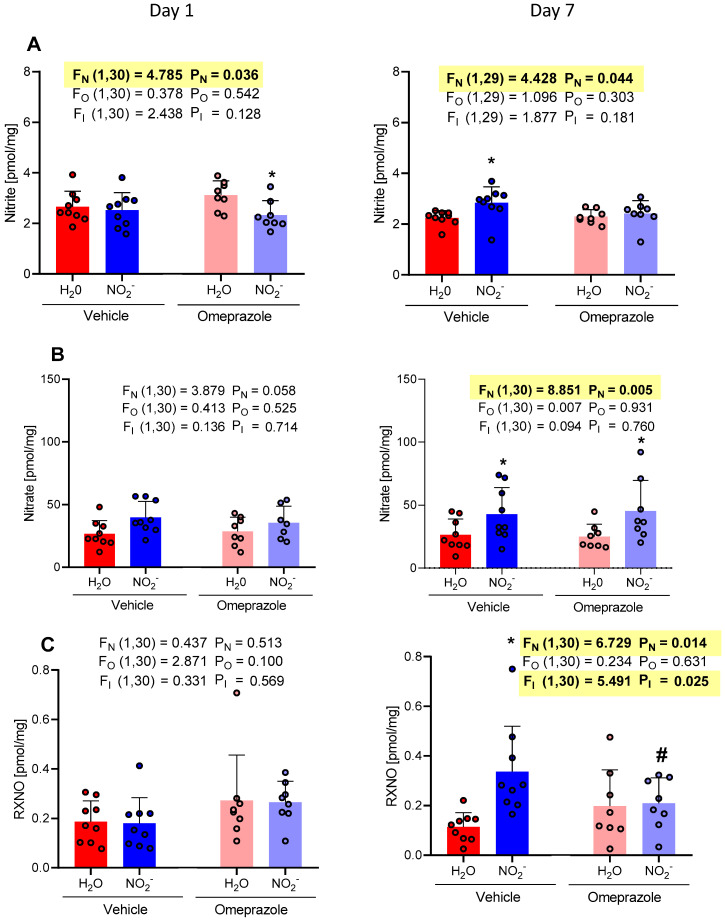
Changes in the concentrations of nitrite, nitrate, and RXNO measured in the heart on day 1 and on day 7 of treatment with sodium nitrite (15 mg/kg) or water via gavage in both vehicle- or omeprazole (10 mg/kg)-treated rats. Blood samples were collected precisely 6 h after the last dose of oral nitrite. (**A**–**C**) display the concentrations of nitrite, nitrate, and RXNO, respectively. Data presented as mean ± S.D. (n = 7–9/group). The values of F correspond to the F-statistics for each factor (F_N_ for nitrite factor, F_O_ for omeprazole factor, and F_I_ for the interaction between factors). * *p* < 0.05 versus respective H_2_O group. # *p* < 0.05 versus vehicle NO_2_^−^ group.

Oral nitrite treatment increased heart concentrations of nitrite, nitrate, and RXNO after 7 days of treatment (Figure 4A, Figure 4B, and Figure 4C, respectively). These findings indicate an accumulation of these metabolites in the heart. Importantly, treatment with omeprazole significantly attenuated RXNO accumulation in the heart after 7 days of oral nitrite treatment (Figure 4C).

On the first day of treatment, the NO_2_^−^ vehicle group exhibited an average nitrite concentration of 2.52 pmol/mg compared to 2.65 pmol/mg in the H_2_O vehicle group. Similarly, the concentrations in the omeprazole NO_2_^−^ group demonstrated an average of 2.32 pmol/mg vs. 3.31 pmol/mg in the omeprazole H_2_O group. By the 7th day of treatment, the NO_2_^−^ vehicle group showed an average concentration of 2.83 pmol/mg vs. 2.24 pmol/mg in the H_2_O vehicle group. The concentrations in the omeprazole NO_2_^−^ group exhibited an average of 2.41 pmol/mg vs. 2.29 pmol/mg in the omeprazole H_2_O group (Figure 4A).

On the 7th day of treatment, the NO_2_^−^ vehicle group presented an average nitrate concentration of 42.87 pmol/mg vs. 26.44 pmol/mg in the H_2_O vehicle group. The concentrations in the omeprazole NO_2_^−^ group demonstrated an average of 45.30 pmol/mg vs. 25.08 pmol/mg in the omeprazole H_2_O group (Figure 4B).

Additionally, the RXNO concentrations on the 7th day of treatment in the NO_2_^−^ vehicle group had an average of 0.33 pmol/mg compared to 0.11 pmol/mg in the H_2_O vehicle group. The concentrations in the omeprazole NO_2_^−^ group exhibited an average of 0.20 pmol/mg vs. 0.19 pmol/mg in the omeprazole H_2_O group (Figure 4C).

### 3.5. Time-Dependent Changes in Gastrocnemius Muscle NO Metabolite Concentrations

**Figure 5:**
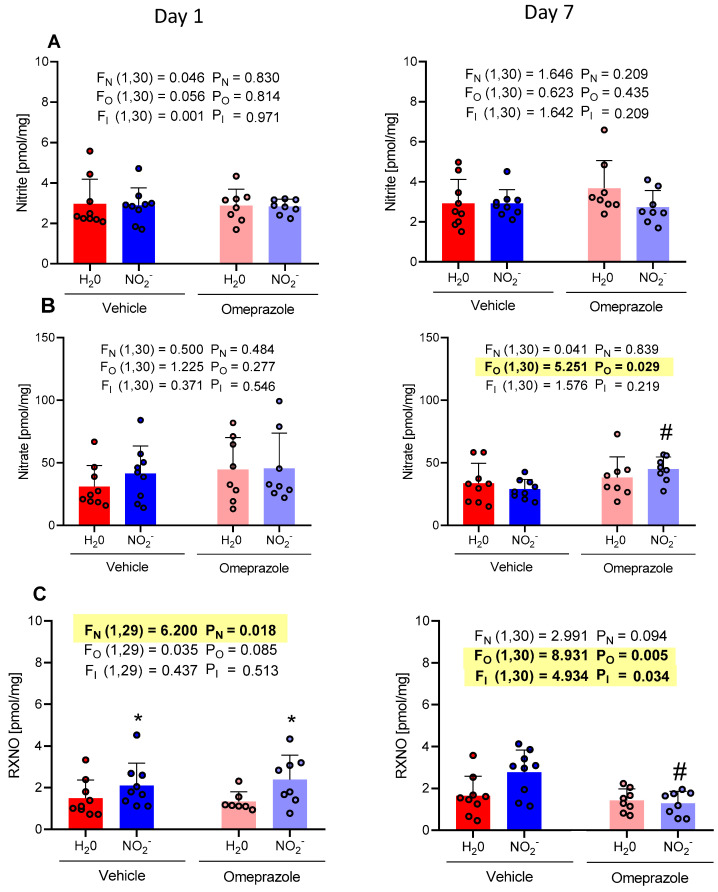
Changes in the concentrations of nitrite, nitrate, and RXNO measured in the gastrocnemius muscle on day 1 and on day 7 of treatment with sodium nitrite (15 mg/kg) or water via gavage in both vehicle- or omeprazole (10 mg/kg)-treated rats. Blood samples were collected precisely 6 h after the last dose of oral nitrite. (**A**–**C**) display the concentrations of nitrite, nitrate, and RXNO, respectively. Data presented as mean ± S.D. (n = 7–9/group). The values of F correspond to the F-statistics for each factor (F_N_ for nitrite factor, F_O_ for omeprazole factor, and F_I_ for the interaction between factors). * *p* < 0.05 versus respective H_2_O group. # *p* < 0.05 versus vehicle NO_2_^−^ group.

Nitrite and nitrate concentrations in the gastrocnemius muscle did not change after 1 or 7 days of oral nitrite treatment (Figure 5A and Figure 5B, respectively). However, RXNO concentrations increased after 1 day of nitrite treatment (Figure 5C). Interestingly, while treatment with omeprazole slightly decreased nitrate concentrations (Figure 5B), it is important to note that omeprazole significantly decreased RXNO concentrations in the muscle, especially after 7 days of treatment with oral nitrite (Figure 5C). These results show that omeprazole attenuated nitrite-induced storage of NO in this muscle as reflected by lower RXNO concentrations measured in this muscle, even after 7 days of nitrite treatment (Figure 5C).

On the 7th day of treatment, the NO_2_^−^ vehicle group presented an average nitrate concentration of 28.90 pmol/mg vs. 33.63 pmol/mg in the H_2_O vehicle group. The concentrations in the omeprazole NO_2_^−^ group demonstrated an average of 44.83 pmol/mg vs. 38.28 pmol/mg in the omeprazole H_2_O group (Figure 5B).

On the first day of treatment, the RXNO concentrations in the NO_2_^−^ vehicle group had an average of 2.10 pmol/mg compared to 1.49 pmol/mg in the H_2_O vehicle group. The concentrations in the omeprazole NO_2_^−^ group exhibited an average of 2.38 pmol/mg vs. 1.33 pmol/mg in the omeprazole H_2_O group. Additionally, on the 7th day of treatment, the NO_2_^−^ vehicle group showed an average RXNO concentration of 2.76 pmol/mg vs. 1.64 pmol/mg in the H_2_O vehicle group. The concentrations in the omeprazole NO_2_^−^ group demonstrated an average of 1.28 pmol/mg vs. 1.42 pmol/mg in the omeprazole H_2_O group (Figure 5C).

### 3.6. Time-Dependent Changes in Liver NO Metabolite Concentrations

**Figure 6:**
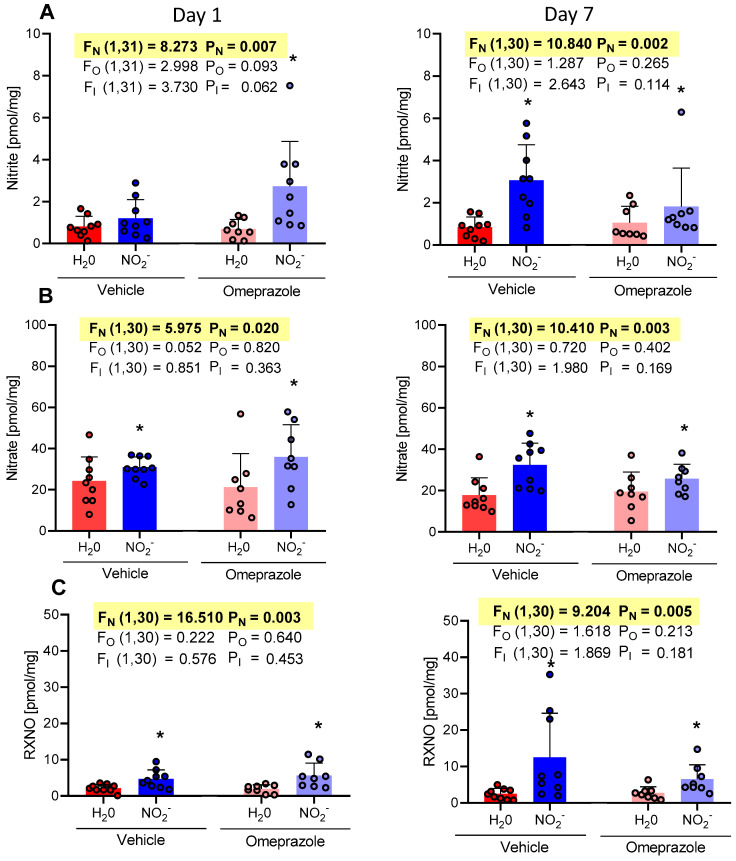
Changes in the concentrations of nitrite, nitrate, and RXNO measured in the liver on day 1 and on day 7 of treatment with sodium nitrite (15 mg/kg) or water via gavage in both vehicle- or omeprazole (10 mg/kg)-treated rats. Blood samples were collected precisely 6 h after the last dose of oral nitrite. (**A**–**C**) display the concentrations of nitrite, nitrate, and RXNO, respectively. Data presented as mean ± S.D. (n = 7–9/group). The values of F correspond to the F-statistics for each factor (F_N_ for nitrite factor, F_O_ for omeprazole factor, and F_I_ for the interaction between factors). * *p* < 0.05 versus respective H_2_O group.

The concentrations of nitrite, nitrate, and RXNO measured in the liver (Figure 6A, Figure 6B, and Figure 6C, respectively) increased significantly after 1 or 7 days of oral nitrite treatment. These findings strongly suggest that the liver serves as an important reservoir for NO metabolites. Interestingly, treatment with omeprazole did not affect this response to oral nitrite treatment.

On the first day of treatment, the NO_2_^−^ vehicle group exhibited an average nitrite concentration of 1.20 pmol/mg vs. 0.81 pmol/mg in the H_2_O vehicle group. The concentrations in the omeprazole NO_2_^−^ group demonstrated an average of 2.72 pmol/mg vs. 0.69 pmol/mg in the omeprazole H_2_O group. By the 7th day of treatment, the NO_2_^−^ vehicle group showed an average nitrite concentration of 3.06 pmol/mg vs. 0.83 pmol/mg in the H_2_O vehicle group. The concentrations in the omeprazole NO_2_^−^ group exhibited an average of 1.81 pmol/mg vs. 1.06 pmol/mg in the omeprazole H_2_O group (Figure 6A).

On the first day of treatment, the nitrate concentrations in the NO_2_^−^ vehicle group had an average of 39.09 pmol/mg compared to 24.24 pmol/mg in the H_2_O vehicle group. The concentrations in the omeprazole NO_2_^−^ group demonstrated an average of 35.93 pmol/mg vs. 21.20 pmol/mg in the omeprazole H_2_O group. Additionally, on the 7th day of treatment, the NO_2_^−^ vehicle group presented an average nitrate concentration of 32.28 pmol/mg vs. 17.76 pmol/mg in the H_2_O vehicle group. The concentrations in the omeprazole NO_2_^−^ group demonstrated an average of 25.68 pmol/mg vs. 19.51 pmol/mg in the omeprazole H_2_O group (Figure 6B).

Moreover, on the first day of treatment, the RXNO concentrations in the NO_2_^−^ vehicle group had an average of 4.69 pmol/mg vs. 2.16 pmol/mg in the H_2_O vehicle group. The concentrations in the omeprazole NO_2_^−^ group exhibited an average of 2.28 pmol/mg vs. 1.94 pmol/mg in the omeprazole H_2_O group. Additionally, on the 7th day of treatment, the NO_2_^−^ vehicle group showed an average RXNO concentration of 12.52 pmol/mg vs. 0.83 pmol/mg in the H_2_O vehicle group. The concentrations in the omeprazole NO_2_^−^ group demonstrated an average of 6.51 pmol/mg vs. 2.71 pmol/mg in the omeprazole H_2_O group (Figure 6C).

## 4. Discussion

It is highly expected that the NO-related species studied here (nitrite, nitrate, and RXNO) accumulate in different tissues after the administration of nitrate or nitrite. However, this is the first study to examine the modifications in tissue concentrations of these metabolites after repeated oral nitrite administration for seven days. In addition, although omeprazole is known to inhibit endogenous NO formation [21] and to critically affect the gastric formation of S-nitrosothiols after oral nitrite administration [6], no previous study has examined whether omeprazole affects the tissue accumulation of the NO-related species studied here. Our findings provide evidence that treatment with oral nitrite promotes increased NO metabolite concentrations, especially in the skeletal muscle and in the liver, with some metabolites also accumulating in the brain and in the heart. These findings may have several pharmacological and clinical implications.

Treatment with nitrite for seven days caused consistent increases in plasma nitrite, nitrate, and RXNO concentrations, even taking into consideration that blood samples were collected 6 h after the last dosing of oral nitrite. This is relevant because previous studies [12] showed that plasma concentrations of nitrite and RXNO returned to baseline levels in less than 2–4 h after a single dose of oral nitrite. Our results, therefore, reinforce the idea that chronic treatment with nitrite could favor the prolonged maintenance of these circulating plasma concentrations of NO metabolites. The mechanisms explaining the changes in tissue concentrations of NO metabolites reported here involve a variety of possible chemical reactions. In fact, our study does not provide clear mechanisms to explain our findings and the chemical reactions taking place in vivo are complex, particularly after the oral administration of nitrite. However, the increases in nitrate concentrations after nitrite administration may be explained by the rapid conversion of nitrite in the blood into nitrate [1,26].

Our results indicate that the aorta does not exhibit significant increases in concentrations of NO metabolites over time following chronic treatment with oral sodium nitrite. These findings contrast with previous studies, which suggested that the nitrite or nitrate (but not RXNO) concentrations increase by 6 to 12 h after a single dose of oral nitrite [11,12]. This apparent discrepancy may be explained by significant differences when acute and chronic treatments are compared. It is possible that more chronic exposure of the aorta to increased nitrite concentrations stimulates mechanisms that regulate NO metabolite concentrations so that they are brought back to normal levels. This possibility remains to be tested and such mechanisms may be more relevant in disease conditions where pathophysiological mechanisms are affected by vascular nitrite and NO concentrations [25,27,28]. There is much complexity when the biology of the interaction of nitrite and other NO-related species is concerned. For example, it is now becoming clear that nitrite may potentiate the vascular responses to S-nitrosothiols [29] via mechanisms that are not known [30] and may involve chemical interactions between nitrite and other species. Moreover, nitrate has been shown to attenuate biological effects of nitrite in the vessels, both in vivo and in vitro [31], and therefore the vascular concentrations of these NO-related species are very importantly and finely regulated.

Intriguingly, we observed that there was a transient increase in brain nitrate and RXNO concentrations on day 1 of oral nitrite treatment, but not after seven days of treatment. These findings are consistent with previous results [12,32], indicating acute increases in brain NO metabolite concentrations followed by a gradual return to baseline levels. The brain, being a highly vascularized organ, handles exposure to exogenous nitrite in a way that may prevent the accumulation of these NO metabolites. This ability may underlie the extensively regulated blood flow in the brain, ensuring a constant supply of oxygen and nutrients. Consistent with this suggestion, previous studies [33,34] suggested that nitrite present in cerebral circulation serves as a source of NO, capable of compensating for compromised NOS function.

Treatment with oral nitrite for seven days increased the heart concentrations of nitrite, nitrate, and RXNO in the present study. These results corroborate previous findings [11,12], which showed increased concentrations of these NO metabolites acutely after a single dose of oral nitrite. This strengthens the idea that nitrite supplementation raises both plasma and cardiac nitrite concentrations, potentially contributing to protection against ischemia/reperfusion injury, possibly attenuating the cardiac damage associated with myocardial infarction [35,36,37,38]. Interestingly, treatment with omeprazole attenuated oral-nitrite-induced increases in RXNO concentrations in the heart, which is consistent with the omeprazole-induced attenuation of gastric formation of nitrosylated species, as previously shown [6]. These findings may help to explain why patients taking omeprazole or other proton pump inhibitors may be exposed to an increased cardiovascular risk [6,20,21].

We found no significant increase in both nitrite and nitrate concentrations in the gastrocnemius muscle after one or seven days of oral nitrite treatment. While treatment with nitrate (not nitrite) has previously shown to result in increased nitrate concentrations in the skeletal muscle [39,40], it should be clear that nitrate is a much more stable NO metabolite, which requires much more complex bioactivation to generate nitrite or NO, and therefore our results cannot be directly compared with those reported in studies using nitrate. However, it is interesting to note that treatment with oral nitrite significantly increased RXNO concentrations in the muscle at day one of treatment, and tended to increase RXNO at day seven of nitrite treatment, thus indicating that the skeletal muscle accumulates RXNO when nitrite is orally administered. Interestingly, treatment with omeprazole blunted this effect. This finding in skeletal muscle parallels the findings in the heart and suggests that omeprazole critically interferes with the oral-nitrite-induced formation of nitrosylated species, resulting in lower RXNO concentrations both in the heart and in the skeletal muscle. This mechanism associated with omeprazole may contribute to many deleterious effects induced with this drug [22] and suggests that omeprazole attenuates or prevents NO storage in both cardiac and skeletal muscle.

The oral administration of sodium nitrite resulted in increased liver nitrite, nitrate, and RXNO concentrations, both on day one and seven of treatment. This is very important because a previous study showed that a single dose of oral nitrite could only transiently increase the liver concentrations of NO metabolites [12], which returned to baseline levels 1–4 h after nitrite administration. The present findings suggest that the liver may accumulate NO metabolites, and therefore the liver may serve as an important reservoir for NO metabolites, which is not affected by the treatment with omeprazole. Further studies are required to explain the mechanisms involved in the accumulation of these metabolites in the liver, as well as the possible benefits of such an effect, particularly in liver diseases.

## 5. Conclusions

The present findings are consistent with the idea that oral nitrite treatment promotes NO metabolite accumulation in different organs, with the skeletal muscle, the heart, and the liver significantly accumulating NO metabolites, particularly RXNO. These responses to oral nitrite are significantly attenuated by omeprazole, particularly in the heart and in the skeletal muscle. The results reported here may indicate that the skeletal muscle, the heart, and the liver are major reservoir sites for NO metabolites after oral nitrite treatment. Our findings may have several clinical implications, particularly to patients with any of the various disease conditions associated with NO depletion. Moreover, our findings may help to explain deleterious effects found in patients taking omeprazole or other proton pump inhibitors.

## Data Availability

Data are contained within the article.
